# Regional Disparities in the Distribution of Healthcare Workers: Evidence From Iran, Chaharmahal and Bakhtiari Province

**DOI:** 10.5539/gjhs.v7n2p374

**Published:** 2015-02-09

**Authors:** Mohamad Ezati Asar, Ramin Varehzardi, Ghasem Rajabi Vasokolaei, Mehdi Haghi, Morteza Fazelipor

**Affiliations:** 1 Research Center for Modeling in Health, Institute for Futures Studies in Health, Kerman University of Medical Sciences, Kerman, Iran; 2 Department of Health Management and Economics, School of Public Health, Tehran University of Medical Sciences, Tehran, Iran; 3 Department of Geology, School of Basic Sciences, Payame Noor University of Abhar-Zanjan, Zanjan, Iran; 4 School of Public Health, Fasa University of Medical Sciences, Fasa, Iran; 5 Department of Health Care Management, School of Allied Medical Sciences, Tehran University of Medical Sciences, Tehran, Iran; 6 Research Center for Health Services Management, Institute for Futures Studies in Health, Kerman University of Medical Sciences, Kerman, Iran; 7 Virology Department, School of Medicine, Ahvaz Jundishapur University of Medical Sciences, Ahvaz, Iran

**Keywords:** regional disparity, distribution, healthcare workers, Iran

## Abstract

A health care service is a prerequisite for sustainable development. This requires access to balanced health workers in different geographic areas. The first step is to identify inequality in access to health workers in different areas. This study is a descriptive study was carried out on the cities in Chaharmahal and Bakhtiari province. TOPSIS technique was used to rank the cities in terms of regional disparities in the distribution of health workers. The findings revealed that distinct disparities in the distribution of healthcare workers across Chaharmahal and Bakhtiari province. Shahrekord and Ardal cities were classified as 1^st^ and 7^th^ respectively. Policy makers should consider priority (regional planning, budget and resources allocation) according to the distribution of healthcare workers.

## 1. Introduction

A well-adjusted distribution of human resources is a step towards elimination of regional disparity (Shaykh Baygloo, 2012). Development and health are strictly associated to each other (Rafi’iyaan & Taajdaar, 2008). Health as an essential community part of national and international level plays a significant role in the welfare of individuals (Sayemiri & Sayemiri, 2001).

The studies have been mostly conducted on the use of economic and social indices and infrastructure development indices to classify different geographical areas, in terms of access to the indices (Poor Fathi & Asheri, 2010). Previously GDP and GDP per capita were used for assessing the development level. These indices do not reflect equality in the distribution of social services and healthcare services (Mahmoudi, 2011). Health as an significant social section of any nation plays a decisive function in the welfare of societies (Sayemiri & Sayemiri, 2001). Access to healthcare is a multi-dimensional concept (Paez, Mercado, Farber, Morency, & Roorda, 2010). This issue has been concern of public policy makers (Amaral, 2009; Comber, Brunsdon, & Radburn, 2011). Researches in most of regions demonstrated that particular regions have enjoyed the new services and facilities (Amini, Yadolahi, & Inanlu, 2006). Islamic Revolution exclusive consideration has been paid to the healthcare section (Ahmadi, Ghaffari, & Emadi, 2011).

Distribution of healthcare indices in the provinces and towns of Iran is heterogeneous (Taghvaei & Shahivandi, 2011). Similar to low and middle income countries, certain regions compared to other regions are accountable for the create national income (Mohammadi, Abdoli, & Fathi Biranvand, 2012). Hence, it is essential to describe the terms of equal distribution of healthcare facilities and designing an integrated plan to fix this problem (Anjomshoa & Mousavi, 2014).

This study was conducted using TOPSIS technique to assess the health workers access across the Chaharmahal and Bakhtiari province. Due to the multiplicity of criteria for comparison subjects, the use of other techniques will lead to problems in decision-making. However, these things do not occur in the TOPSIS technique. TOPSIS technique as a family member in a Multiple Criteria Decision Making (MCDM) are used to rank the various concepts of knowledge, due to the transparent nature, mathematical logic and haven’t any operational issues (Zyaree, Zanjirchee, & Sorkhkamal, 2010). The aim of this study is to provide a clear vision from the status of Chaharmahal and Bakhtiari cities in terms of distribution of health workers.

## 2. Methods

This was a descriptive study for health system evaluation. The sample included all 7 towns of Chaharmahal and Bakhtiari province that is located at the center of Zagros Mountains. This province is limited to Isfahan from the east and the north, to Khuzestan province from the west, to Kohkilooyeh and Boyer Ahmad from the south and to Lorestan province from the Northwest. The city of Shahrekord is the center of Chaharmahal and Bakhtiari province. According to the SCI data, the growth rate (average annual) of the population of Chaharmahal and Bakhtiari is 0.86 have been calculated and its population percentage is 1.19. The numbers of 5 health workers indices were selected based on their availability at 2011 National Statistics Center’s annual report as follows (Table 1).

**Table 1:** Indices to determine the Regional disparities in the distribution of healthcare workers

Code	Indices
**1**	Number of general practitioner per 1000 people
**2**	Number of rural active health house workers per 1000 people
**3**	Number of pharmacologist per 10 thousand people
**4**	Number of specialist per 1000 people
**5**	Number of paramedical per 1000 people

To assessing the development level, several quantitative techniques are available which depending the validity and reliability of the existing evidence and the skills of local authorities are employed to plan, organize and evaluate past information (Badri & Akbarian Ronizi, 2007). One method of grading the areas according to facility distribution is the TOPSIS technique. TOPSIS technique has gained significance as one of the MCDM techniques to rank the different concepts of numerous branches of science, the most important reason being it’s clear, mathematical logic and the practical problems associated with it. TOPSIS technique is done in the following stages (Zyaree, Mohamadi, & Atar, 2012):

1) -The maximum 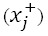 and minimum 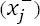 values of each index are recognized. Then, using the following equation, normalization takes place. If the positive and negative indices are intended to be combined reversing the negative aspects into positive aspects should be done as follows:
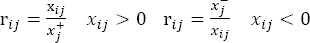
2) -The standard weighted matrix based on the following equation is attained:
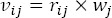

The positive ideal and negative ideal solutions for each of the indices are determined by the following formula:


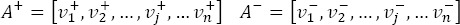


The positive ideal index is equal to its maximum, and the negative ideal in every index, the index is equal to the minimum. Separation of each option compared with ideals of positive and negative are as follows (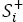,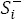):

Separation from the negative ideal solution 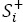:


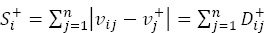


Separation from the negative ideal solution 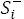:


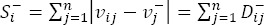


For calculation of relative closeness of each alternative to the ideal we should combine the values of 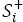 and 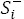:


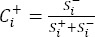


The classifying is done based on the decreasing values of 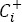, means that the highest 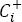 is considered as the most developed, and the lowest 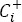 as most Underdeveloped.

## 3. Results

The aim of the current paper is to describe the regional disparities in the distribution of healthcare workers in Chaharmahal and Bakhtiari cities. Chaharmahal and Bakhtiari towns were compared in terms of access to healthcare workers using TOPSIS technique. To offer a clear vision from the status of Chaharmahal and Bakhtiari cities, 7 towns were measured and ranked into three classified (Table 1).

**Table 1:** Chaharmahal and Bakhtiari cities ranking in terms of access to healthcare workers

Rank the preference order	City name	Separation from the negative ideal solution $$$	Separation from the positive ideal solution $$$	TOPSIS Index Ci+	Degree of development
**1**	Shahrekord	0.358	1.105	0.756	
**2**	Borujen	0.439	0.861	0.662	Developed
**3**	Farsan	0.608	0.693	0.532	
**4**	Koohrang	0.993	0.701	0.414	Developing
**5**	Kiar	0.976	0.432	0.307	
**6**	Lordegan	0.983	0.391	0.284	Underdeveloped
**7**	Ardal	1.152	0.133	0.3104	

The results show that the highest and lowest degrees of development based on TOPSIS technique in Chaharmahal and Bakhtiari province, belonged to Shahrekord (0.756) and Ardal (0.104) respectively. Findings show that in terms of access to healthcare workers, Shahrekord, Borujen and Farsan can be regarded as developed, while Koohrang and Kiar are categorized as developing. Lordegan and Ardal can be regarded as underdeveloped. The findings illustrate a gap between Chaharmahal and Bakhtiari towns.

## 4. Discussion

Accessibility of healthcare services is one of the most important indicators for developing countries (Zarrabi & Shaykh Baygloo, 2011). The public scarcity of healthcare workers in low-middle income countries, there is a general understanding that enormous in country disparities exist in the distribution of healthcare workers (Munga & Maestad, 2009). In this study we tried to examine the regional disparities in healthcare workers across Chaharmahal and Bakhtiari province. 5 indices were selected and analyzed using TOPSIS technique.

Healthcare workers should be accessible for all people in the Farthest and poorest parts of the country; however some services, such as specialist only delivered at larger cities and cover the suburbs. Smaller cities with a population lower than the service for population threshold only can be provided with mobile services or only on special weekdays (Zarrabi & Shaykh Baygloo, 2011).

The results of this study show that access to healthcare workers was very different among people in the Chaharmahal and Bakhtiari province. 42.8 % of the cities (Shahrekord, Borujen and Farsan) were in the level of development; 28.6 % (including two cities: Koohrang and Kiar) were in developing level, and 28.6 of cities (including two cities: Lordegan and Ardal) were underdeveloped in terms of their access to healthcare workers.

Shahrekord took the most and Ardal the least access to the healthcare workers. In Iran studies such as Kurdistan province (Zangi Abadi, AmirAzodi, & Parizadi, 2012), Isfahan province (Nastaran, 2001; Zarabi, Mohammadi, & Rakhshani Nasab, 2008), Markazi province (Abolhallaje et al., 2014), Mashhad metropolis (Rafi’iyaan & Taajdaar, 2008), Kerman province (Anjomshoa et al., 2014), Kermanshah province (Mousavi et al., 2013) and Bushehr province (Sadeghifar et al., 2014) were similar in conclusions about regional disparities in the health services access. In other countries (Anand et al., 2008; Gravelle, & Sutton, 2001; Munga, & Maestad, 2009; Sousa, Dal Poz, & Carvalho, 2012; Theodorakis, & Mantzavinis, 2005) similar results have been obtained in terms of access to healthcare workers.

## 5. Conclusion

In order to decrease gap of access to health workers between the cities and equitable distribution, concentration on development of health indicators in poor cities (such as Lordegan and Ardal), is recommended. Furthermore, it is suggested that in the first stages of city development, authorities need to focus on short term policies and programs which could result in expansion of critical health workers and equity in access and authorities may pay attention to the development of necessary services in developing and deprived cities over a medium and long term plan (5-10 years).
